# Health-promoting lifestyles among nursing students from Argentina

**DOI:** 10.15649/cuidarte.4614

**Published:** 2025-08-05

**Authors:** Carlos Jesús Canova-Barrios, María Alejandra Mangano, Sara Leonor Mercado

**Affiliations:** 1 Universidad de Ciencias Empresariales y Sociales (UCES), Ciudad Autónoma de Buenos Aires, Argentina. E-mail: carlos.canova1993@gmail.com UCES Buenos Aires Argentina carlos.canova1993@gmail.com; 2 Universidad Nacional del Sur & Universidad Provincial del Sudoeste, Bahía Blanca, Argentina. Email: alejandra.mangano@upso.edu.ar Universidad Nacional del Sur & Universidad Provincial del Sudoeste Bahía Blanca Argentina alejandra.mangano@upso.edu.ar; 3 Universidad Nacional del Sur & Universidad Provincial del Sudoeste, Bahía Blanca, Argentina. Email: saralmercado@yahoo.com.ar Universidad Nacional del Sur & Universidad Provincial del Sudoeste Bahía Blanca Argentina saralmercado@yahoo.com.ar

**Keywords:** Healthy Lifestyle, Students, Nursing, Self Care, Health Promotion, Education, Nursing, Estilo de Vida Saludable, Estudiantes, Enfermería, Autocuidado, Promoción de la Salud, Educación en Enfermería, Estilo de Vida Saudável, Estudantes, Enfermagem, Autocuidado, Promoção da Saúde, Educação em Enfermagem

## Abstract

**Introduction:**

Lifestyles comprise the set of habits and behaviors that influence individuals’ health and well-being. When not properly adopted, they can contribute to the development of chronic diseases and a decline in quality of life.

**Objective:**

To describe health-promoting lifestyles among nursing students at a public university in the Province of Buenos Aires, Argentina, in 2024.

**Materials and Methods:**

An analytical, cross-sectional, quantitative study was conducted. The study included 314 nursing students who completed Nola Pender’s Health-Promoting Lifestyle Profile - II. For the inferential analysis, Student’s t-tests, ANOVA, and Pearson’s correlation test were applied.

**Results:**

Respondents had a mean age of 27.04 years (SD = 8.11); most were female (86.62%), single (76.43%), childless (73.25%), working (55.41%), and enrolled in the fourth year of the program (24.52%). Healthy ratings were observed in the dimensions of Spiritual growth and Interpersonal Relations, while the remaining dimensions were rated as unhealthy. The average lifestyle score was 124.97 points (SD = 20.49), equivalent to 60.08% of the instrument’s total score. A total of 78.66% of respondents had a lifestyle categorized as regular.

**Discussion:**

It is necessary to implement interventions aimed at strengthening self-care practices among nursing students and identify the associated factors.

**Conclusion:**

Most of the respondents exhibited a regular lifestyle with high levels of physical inactivity, overweight, poor dietary habits, and inadequate stress management.

## Resumen

**Introducción:**

Los estilos de vida son el conjunto de hábitos y costumbres que influyen en la salud y bienestar de las personas; no aplicarlos adecuadamente puede llevar al desarrollo de enfermedades crónicas y un deterioro en la calidad de vida.

**Objetivo:**

Describir los estilos de vida promotores de salud en estudiantes de Enfermería de una universidad pública en la provincia de Buenos Aires, Argentina en 2024.

**Materiales y métodos:**

Estudio analítico, transversal y cuantitativo. Participaron 314 estudiantes de Enfermería quienes completaron el instrumento Health-Promoting Lifestyle Profile – II de Nola Pender. Para el análisis inferencial se aplicaron las pruebas T de Student, ANOVA y prueba de correlación de Pearson.

**Resultados:**

Los encuestados tuvieron una edad promedio de 27,04 años (DE: 8,11), y en su mayoría pertenecen al género femenino (86,62%), solteros (76,43%), sin hijos (73,25%), laboralmente activos (55,41%) y cursando el cuarto año del plan de estudios (24,52%). Se encontró una valoración saludable en las dimensiones Autorrealización y Relaciones Interpersonales, y poco saludable en las demás. El estilo de vida obtuvo un puntaje promedio de 124,97 puntos (DE=20,49) equivalente al 60,08% del puntaje total del instrumento. El 78,66% de los encuestados presentaron un estilo de vida categorizado como regular.

**Discusión:**

Se requiere implementar intervenciones orientadas a fortalecer las prácticas de autocuidado en los estudiantes de Enfermería e identificar los factores relacionados.

**Conclusiones:**

En su mayoría los encuestados presentaron un estilo de vida regular con altos índices de inactividad física, sobrepeso, hábitos de alimentación deficientes e inadecuado manejo del estrés.

## Resumo

**Introdução:**

Estilos de vida são o conjunto de hábitos e costumes que influenciam a saúde e o bem-estar das pessoas; a não implementação adequada pode levar ao desenvolvimento de doenças crônicas e à deterioração da qualidade de vida.

**Objetivo:**

Descrever os estilos de vida promotores da saúde de estudantes de enfermagem de uma universidade pública na província de Buenos Aires, Argentina, em 2024.

**Materiais e métodos:**

Estudo analítico, transversal e quantitativo. Participaram 314 estudantes de enfermagem que preencheram o Perfil de Estilo de Vida Promotor da Saúde – II de Nola Pender. Testes T de Student, ANOVA e correlação de Pearson foram utilizados para análise inferencial.

**Resultados:**

Os entrevistados tinham idade média de 27.04 anos (DP: 8.11), sendo a maioria do sexo feminino (86.62%), solteira (76.43%), sem filhos (73.25%), empregada (55.41%) e cursando o quarto ano de estudo (24.52%). Foi encontrada avaliação saudável nas dimensões Autorrealização e Relações Interpessoais, e não saudável nas demais. O estilo de vida obteve escore médio de 124.97 pontos (DP = 20.49), equivalente a 60.08% do escore total do instrumento. 78.66% dos respondentes apresentaram estilo de vida categorizado como regular.

**Discussão:**

Faz-se necessária a implementação de intervenções que visem ao fortalecimento das práticas de autocuidado em estudantes de enfermagem e à identificação dos fatores relacionados.

**Conclusões:**

A maioria dos respondentes apresentou estilo de vida regular, com altos índices de inatividade física, sobrepeso, maus hábitos alimentares e manejo inadequado do estresse.

## Introduction

The World Health Organization (WHO)^1^ defines health as “a state of complete physical, mental, and social well-being and not merely the absence of disease or infirmity.” Maintaining an optimal state of health depends on various factors grouped under the concept of lifestyle (LS), which includes a balanced diet, adequate rest, regular physical activity, health monitoring and control, abstaining from tobacco and drug use, and low or no alcohol consumption, among others^2^. A LS is considered health-promoting when it comprises habits and practices that support physical, mental, and social well-being. Conversely, an inadequate lifestyle is considered a risk factor for the development of chronic noncommunicable cardiometabolic diseases that adversely affect individuals' quality of life and well-being^3^,^4^. 

Academic life has been identified as a factor that significantly impacts the maintenance of a healthy LS, as it often limits the time available for proper nutrition, physical activity, rest, and self-care^5^. In the case of students in health-related disciplines, they acquire knowledge intended to encourage health-promoting behaviors in their patients. However, it has been observed that these habits are frequently not applied in their own daily lives, negatively affecting their academic performance and overall well-being^6^-^11^. 

In Argentina, the curriculum of the Bachelor's Degree in Nursing, declared a degree of public interest since 2013, has specific characteristics, including a heavy academic workload and high demands (of time and effort) in hospital and community practice settings. The nursing program has a minimum duration of four years, often extending to five years in public higher education institutions. The curriculum is organized into two cycles: upon completing the third year, students obtain the title of Nurse, and at the end of the final year (fourth or fifth, depending on the institution), they obtain the Bachelor of Science in Nursing degree. These features are relevant when analyzing self-care patterns within this population. Furthermore, the complex socioeconomic context of the Argentine Republic, marked by high inflation rates and steadily declining purchasing power, prompts students to take on heavy workloads to earn an income that allows them to meet their basic needs while keeping up with the academic demands of the program, affecting the time available for self-care. In recent years, there has been a growing body of research focused on describing the LS of nursing students, revealing a high prevalence of risk factors for both communicable and non-communicable diseases. In Argentina, the 4th National Survey of Risk Factors (ENFR, by its acronym in Spanish)^12^ reported a high prevalence of risk factors, such as smoking (22.2%), overweight and obesity (66.1%), excessive alcohol consumption (13.3%), and low intake of fruits and vegetables (6.0%). A multicenter study conducted in Argentina^5^, which included 1,718 students from eight higher education institutions, identified a high level of unhealthy habits, with 68.92% of students exhibiting what was categorized as a “regular” LS. Similar findings have been corroborated in other studies involving comparable populations^13^,^14^. 

Nola Pender, the author of the Health Promotion Model, addresses in her theory aspects related to individuals’ decisions to adopt self-care behaviors. These aspects include expectations about one’s health status, knowledge, beliefs, and previous experiences regarding the adoption of habits associated with a healthy LS^10^. This theorist proposes four conditions for the adoption of health-promoting LS: attention (interest in the observed habits), retention (the ability to remember what has been learned), reproduction (the ability to replicate the observed behaviors), and motivation (the elements that drive the implementation of the habit). Based on this, it would be expected that students, particularly those enrolled in nursing programs, would incorporate the health-promoting behaviors they learn into their own daily lives. However, the evidence reported in the literature is contrasting^5^,^11^,^13^,^14^. 

It is therefore necessary to conduct studies that evaluate the extent to which nursing students implement health-promoting LS, as well as the factors associated with their implementation, in order to design intervention plans aimed at their improvement. 

Based on the elements previously outlined, the present study aimed to describe health-promoting lifestyles among students enrolled in the Bachelor’s Degree in Nursing at a public university in the province of Buenos Aires, Argentina, during the first four-month period of 2024. 

## Materials and Methods

A quantitative, analytical, and cross-sectional study was conducted at a public higher education institution in the city of Bahía Blanca, Province of Buenos Aires (Argentina), during the first four-month period of 2024. The target population consisted of 609 students enrolled in the Bachelor of Science in Nursing program. A sample of 314 students (51.55% of the universe) was selected through non-probabilistic convenience sampling. This sample ensured a 95% confidence level, a margin of error of less than 5%, and adequate representation of students from all years of the nursing program. The randomization process was supported by the Working in Epidemiology tool (WinEpi ©2006). 

Students enrolled and actively attending from the first to the fifth year of the Bachelor of Science in Nursing program who voluntarily agreed to participate by signing the informed consent form were included in the study. Forms that were incorrectly completed or had missing data were excluded. 

To collect information, the Health Promoting Lifestyle Profile II (HPLP-II), an instrument developed by Nola Pender under her Health Promotion Model, was used. This tool has a reported Cronbach’s alpha ranging from 0.70 to 0.93^5^,^11^,^14^. The instrument consists of 52 items grouped into six dimensions: Health Responsibility, Nutrition, Spiritual growth, and Interpersonal Relations (nine items each); and Physical Activity and Stress Management (eight items each). Responses are recorded on a four-point Likert scale: Never (1 point), Sometimes (2 points), Often (3 points), and Routinely (4 points). The total score of the 52 items allows for categorizing the lifestyle as unhealthy (52–104 points), regular (105–156 points), or healthy (157–208 points). Likewise, each dimension is also classified as healthy (≥61% of the maximum score for that dimension) or unhealthy (<60%) (see Table 1). 

**Table 1:** Characteristics of lifestyle dimension scores

Dimension	Items	Unhealthy	Healthy
Health responsibility	3, 9, 15, 21, 27, 33, 39, 45, and 51	9 to 22	23 to 36
Physical activity	4, 10, 16, 22, 28, 34, 40, and 46	8 to 19	20 to 32
Nutrition	2, 8, 14, 20, 26, 32, 38, 44, and 50	9 to 22	23 to 36
Spiritual growth	6, 12, 18, 24, 30, 36, 42, 48, and 52	9 to 22	23 to 36
Interpersonal relations	1, 7, 13, 19, 25, 31, 37, 43, and 49	9 to 22	23 to 36
Stress management	5, 11, 17, 23, 29, 35, 41, and 47	8 to 19	20 to 32

Six questions were included to collect sociodemographic and academic characteristics of the students (age, gender, marital status, number of children, employment status, and year of study), along with Body Mass Index (BMI)^6^. The instrument was uploaded to Google Forms and distributed through the institutional campus platform and participants’ email addresses. 

For the analysis, the data were exported into a Microsoft Excel matrix and processed using the Infostat v/L software. Descriptive statistics included absolute and relative frequencies for categorical variables, as well as measures of central tendency and dispersion (mean and standard deviation) for quantitative variables. For inferential analysis, since the variables followed a normal distribution (W = 0.00; p = 0.859), Student’s t-test (for comparing means between two groups), ANOVA (for comparing means across three or more groups), and Pearson’s correlation coefficient (for relationships between quantitative variables) were used. A significance level of p = 0.05 was established. 

The study was approved by the Institutional Research Ethics Committee (Resolution 0032, October 2023). Participation was voluntary, and informed consent was obtained. Data collection ensured participant anonymity, as no identifying personal information was gathered. The full dataset is available for free access and consultation on Mendeley Data^15^. 

## Results

A total of 314 undergraduate nursing students participated, with a mean age of 27.04 years (SD: 8.11). The majority were female (86.62%), single (76.43%), without children (73.25%), working (55.41%), and enrolled in the fourth year of the program (24.52%). The average Body Mass Index (BMI) was 25.86 kg/m² (SD = 6.08), with 49.04% of participants classified as having a normal weight (see Table 2). 

It was found that the item “Attend educational programs on personal health care” from the Health Responsibility dimension had the lowest mean score (1.42; SD = 0.66), while the item “Touch and am touched by people I care about” from the Interpersonal Relations dimension had the highest mean score (3.29; SD = 0.75) (see Table 3). 

**Table 2:** Sample characteristics

Variable	% (n)
Age. Mean (SD)	8.11 (27.04)
Gender	
Female	86.62 (272)
Male	12.42 (39)
Other	0.96 (3)
Marital status	
Single	76.43 (240)
Cohabiting or married	21.34 (67)
Divorced or widowed	2.23 (7)
Children	
Yes	26.75 (84)
No	73.25 (230)
Working	
Yes	55.41 (174)
No	44.59 (140)
Year of study	
First	19.75 (62)
Second	20.38 (64)
Third	22. 93 (72)
Fourth	24.52 (77)
Fifth	12.42 (39)
Body Mass Index	
Underweight	4.78 (15)
Normal weight	49.04 (154)
Overweight	29.94 (94)
Grade 1 obesity	9.55 (30)
Grade 2 obesity	3.82 (12)
Grade 3 obesity	2.87 (9)
Total	100.00 (314)

**Table 3:** Mean and standard deviation of items in the health-promoting lifestyle profile among nursing students

Item	Mean ± SD
Discuss my problems and concerns with people close to me.	2.59 ± 0.75
Choose a diet low in fat, saturated fat, and cholesterol	2.24 ± 0.81
Report any unusual signs or symptoms to a physician or other health professional.	2.41 ± 0.81
Follow a planned exercise program.	2.18 ± 0.99
Get enough sleep.	2.18 ± 0.71
Feel I am growing and changing in positive ways.	2.72 ± 0.75
Praise other people easily for their achievements.	3.15 ± 0.72
Limit use of sugars and food containing sugar (sweets).	2.29 ± 0.93
Read or watch TV programs about improving health.	1.93 ± 0.86
Exercise vigorously for 20 or more minutes at least three times a week (such as brisk walking, bicycling, aerobic dancing, using a stair climber).	2.41 ± 1.06
Take some time for relaxation each day.	2.21 ± 0.74
Believe that my life has purpose.	3.04 ± 0.79
Maintain meaningful and fulfilling relationships with others.	3.08 ± 0.77
Eat 6-11 servings of bread, cereal, rice and pasta each day.	1.79 ± 0.81
Question health professionals in order to understand their instructions.	2.62 ± 0.8
Take part in light to moderate physical activity (such as sustained walking 30-40 minutes 5 or more times a week).	2.32 ± 0.91
Accept those things in my life which I cannot change.	2.59 ± 0.76
Look forward to the future.	3.1 ± 0.73
Spend time with close friends.	2.68 ± 0.79
Eat 2-4 servings of fruit each day.	2.45 ± 0.91
Get a second opinion when I question my health care provider's advice.	2.08 ± 0.78
Take part in leisure-time (recreational) physical activities (such as swimming, dancing, etc.).	1.95 ± 0.92
Concentrate on pleasant thoughts at bedtime.	2.29 ± 0.78
Feel content and at peace with myself.	2.62 ± 0.82
Find it easy to show concern, love and warmth to others.	2.81 ± 0.94
Discuss my health concerns with health professionals.	2.51 ± 0.85
Eat 3-5 servings of vegetables each day.	2.31 ± 0.86
Do stretching exercises at least 3 times per week.	2.12 ± 0.99
Use specific methods to control my stress	1.71 ± 0.84
Work toward long-term goals in my life	2.92 ± 0.74
Touch and am touched by people I care about.	3.29 ± 0.75
Eat 2-3 servings of milk, yogurt, or cheese each day.	2.43 ± 0.89
Inspect my body at least monthly for physical changes/danger signs.	2.52 ± 0.88
Get exercise during usual daily activities (such as walking during lunch or using stairs instead of elevators).	2.29 ± 0.88
Balance time between work and play.	2.19 ± 0.74
Find each day interesting and challenging.	2.42 ± 0.81
Find ways to meet my needs for intimacy.	2.48 ± 0.78
Eat only 2-3 servings from the meat, poultry, fish, dried beans, eggs, and nuts group each day.	2.45 ± 0.84
Ask for information from health professionals about how to take good care of myself.	2.12 ± 0.81
Check my pulse rate when exercising.	1.79 ± 0.95
Practice relaxation or meditation for 15-20 minutes daily.	1.47 ± 0.75
Am aware of what is important to me in life.	3.02 ± 0.73
Get support from a network of caring people.	2.63 ± 0.81
Read labels to identify nutrients, fats, and sodium content in packaged food.	2.01 ± 1.04
Attend educational programs on personal health care	1.42 ± 0.66
Reach my target heart rate when exercising	1.67 ± 0.83
Pace myself to prevent tiredness.	1.87 ± 0.72
Feel connected with some force greater than myself.	2.24 ± 1.09
Settle conflicts with others through discussion and compromise.	2.95 ± 0.76
Eat breakfast.	2.85 ± 1.01
Seek guidance or counseling when necessary.	2.85 ± 0.79
Expose myself to new experiences and challenges.	2.74 ± 0.79

**Figure 1:**
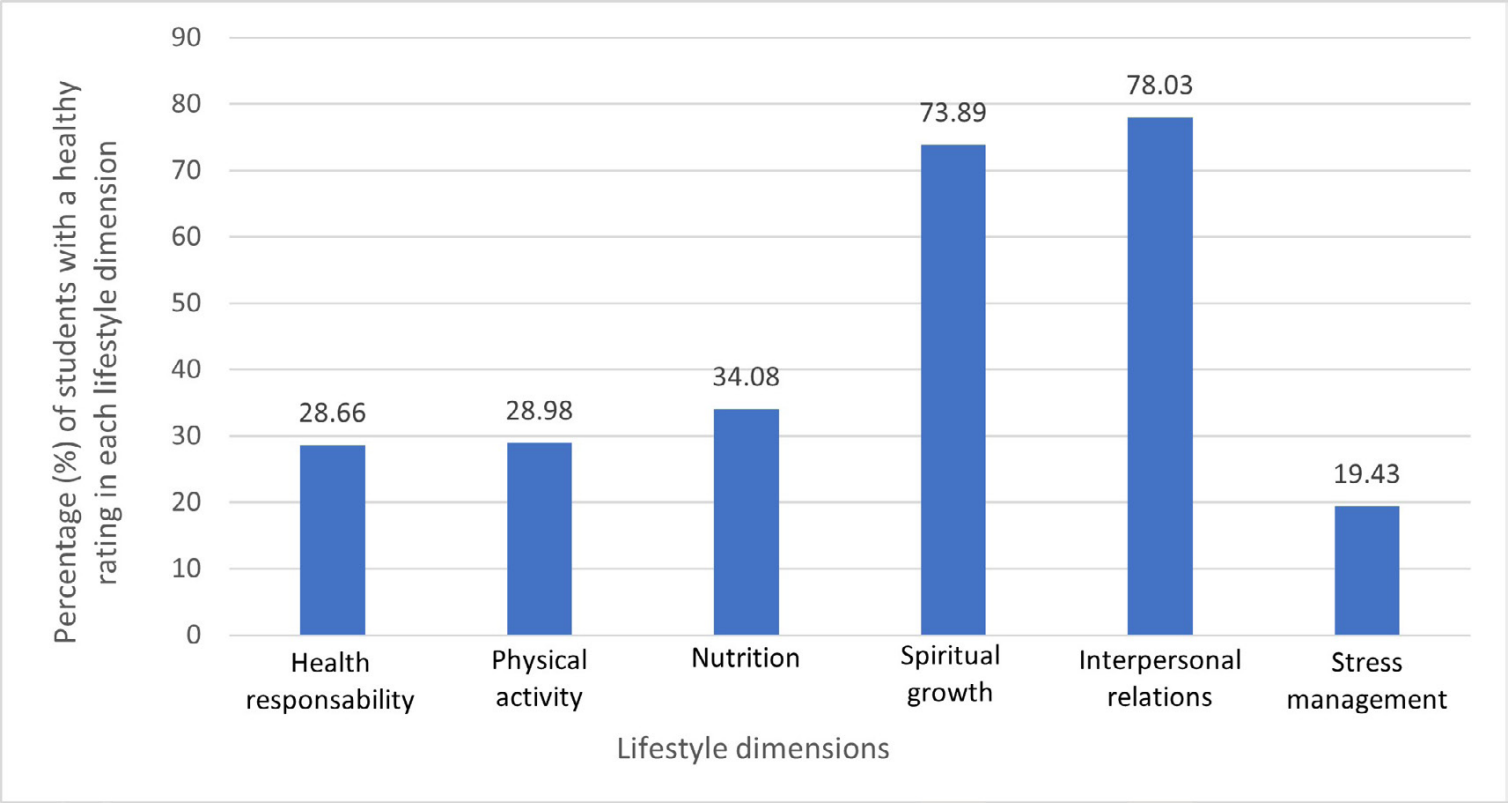
Healthy rating by lifestyle dimensions

When analyzing the dimensions of lifestyle, Interpersonal Relations was the best rated with a mean of 25.66 points (95% CI = 25.15-26.16), and 78.03% of participants were categorized as healthy in this dimension. In contrast, Stress Management showed the lowest scoring with a mean of 16.51 points (95% CI = 16.13-16.88), and only 19.43% of respondents were classified as healthy in this area (see Figure 1). 

**Figure 2:**
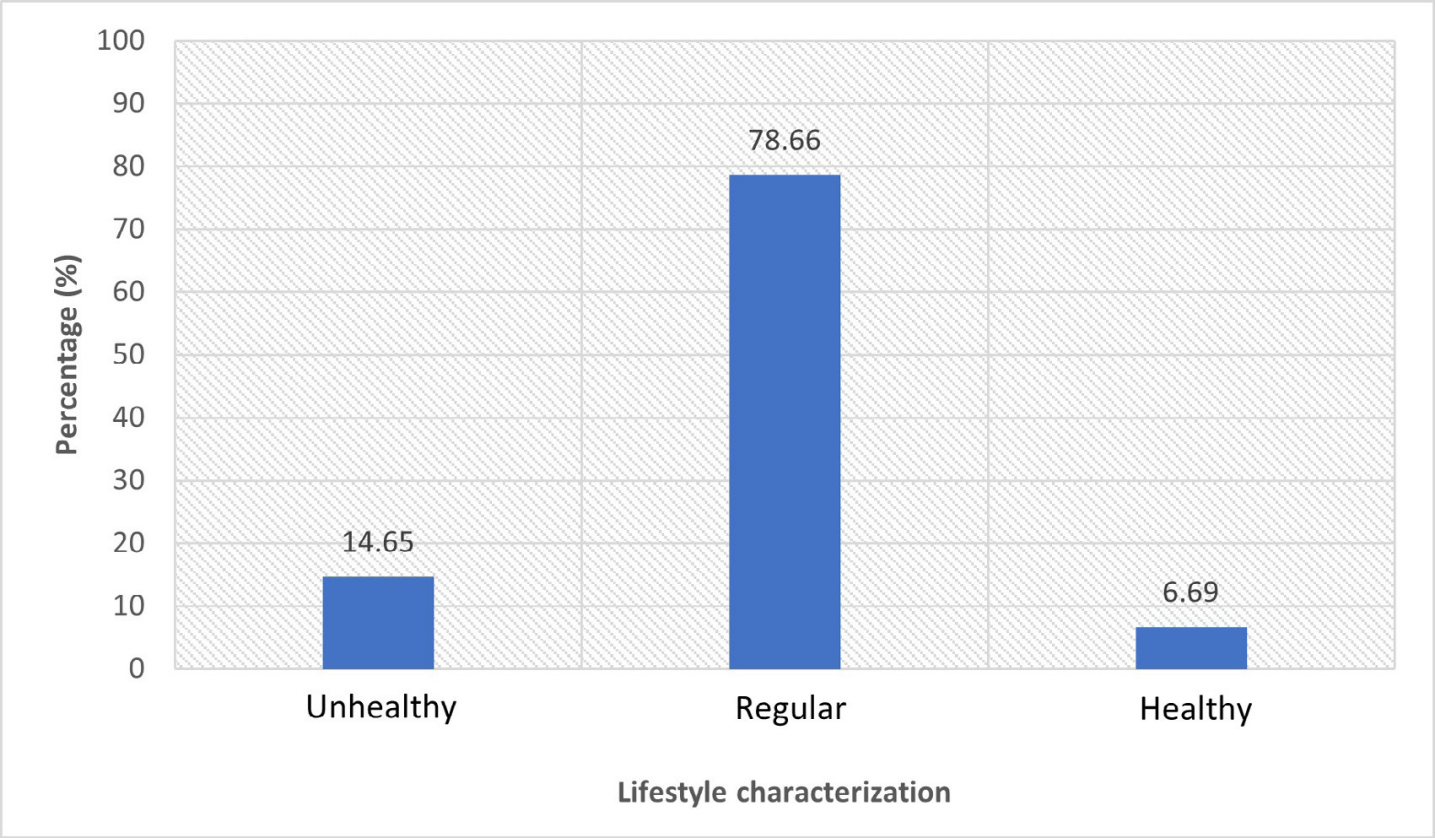
Lifestyle characterization of nursing students

A total of 78.66% (n=247) of the participants presented a lifestyle categorized as regular (see Figure 2), with an overall mean lifestyle score of 124.97 points (SD = 20.49, 95% CI = 122.70-127.25), equivalent to 60.08% of the total possible score on the instrument. 

Inferential analysis revealed a negative correlation between age and the Physical Activity dimension (r = –0.15, p = 0.006), and a positive correlation between age and BMI (r = 0.31, p <0.001). 

When comparing the mean scores of the LS dimensions against students’ sociodemographic and academic variables, the following findings were observed: higher scores (better lifestyle) in the Health Responsibility dimension among female students (p=0.005); higher scores in Physical Activity among students without children (p=0.015), those who were not working (p = 0.024), and those taking third-year courses of the study plan (p<0.001); and better Stress Management among students without children (p=0.023), and those who were not working (p=0.008). Likewise, lower score means in the Nutrition dimension were observed among students taking first-year courses (p=0.038) (see Table 4). The BMI score showed a low, negative correlation with the Nutrition dimension (r=-0.12, p=0.027). 

**Table 4:** Associations between lifestyle dimensions and sociodemographic and educational variables

Categories	HR	PA	NT	SG	IR	SM	LS
Gender							
Female	20.57*	16.56	21.05	24.95	25.86	16.54	125.52
Male	18.38	17.97	20.54	24.38	24.46	16.49	122.23
Marital status							
Single	20.34	17.00	21.00	24.63	25.76	16.65	125.39
Cohabiting or married	20.01	15.94	21.07	25.43	25.46	15.99	123.91
Children							
Yes	20.36	15.55	20.48	25.38	25.4	15.87	123.04
No	20.23	17.14*	21.2	24.62	25.75	16.74*	125.68
Working							
Yes	20.25	16.13	20.72	25.25	25.79	16.05	124.18
No	20.28	17.45*	21.33	24.29	25.49	17.08*	125.96
Year of study							
First	19.15	15.21	19.44*	24.16	25	16.27	119.23
Second	20.39	18.06	21.52	23.95	25	16.72	125.64
Third	20.57	18.49*	21.43	24.51	26.1	17.06	128.15
Fourth	20.82	15.69	21.08	25.78	25.6	16.42	125.38
Fifth	20.18	15.67	21.77	25.97	27.08	15.69	126.36

Note: *Statistically significant values. HR: Health responsibility, PA: Physical activity, NT: Nutrition, SG: Spiritual growth, IR: Interpersonal relations, SM: Stress management, and LS: Lifestyle.

## Discussion

In the present study, the majority of participants were female, single, childless, working, and enrolled in the fourth year of the nursing program. Respondents were found to have a LS classified as regular. Likewise, a healthier LS was observed among female students, those without children, those exclusively dedicated to their studies, and those in the first cycle of the program (first to third year of the study plan).

These data are consistent with previous literature reporting higher levels of health responsibility among women, greater physical activity, and better stress management among students without children and those not working, likely due to greater time availability and fewer competing responsibilities^16^-^19^. At the same time, unhealthy nutritional habits were identified among recently enrolled students in the program, possibly due to a lack of knowledge and awareness about healthy eating.

Age was negatively correlated with the physical activity dimension and positively correlated with BMI. This suggests a decline in physical activity levels with age increase, likely associated with growing responsibilities (such as parenting or a lack of exclusive dedication to studies), which may explain the higher BMI at older ages. While these results contradict other studies reporting higher levels of physical activity among older students^18^,^19^, they are consistent with others that have identified a decrease in health accountability with age^14^.

In the present study, high levels of overweight and obesity were identified among students of the Bachelor's Degree in Nursing. This finding aligns with the high levels of sedentary behavior and poor eating habits reported by nearly three-quarters of participants, consistent with findings from several other studies^20^,^21^. However, it contrasts with reports from other student populations that demonstrated more adequate dietary patterns^22^.

Approximately one-fifth of respondents reported poor stress management, a factor previously associated with the combined demands of academic, social, family, and work responsibilities^23^-^25^. Considering that over half of the students were working, one-quarter had children, and most were women, these circumstances added responsibilities associated with these factors.

Finally, LS was classified as regular in 78.66% of the respondents, which coincides with studies conducted among nursing students, where the regular category was reported in approximately one-sixth to one-seventh of participants^5^,^11^,^14^,^19^,^26^-^28^. Undoubtedly, the demands of higher education, particularly in health-related programs, significantly influence students’ self-care patterns. This underscores the need for designing interventions to sustain and improve students’ lifestyles, thereby preventing health deterioration and the entrenchment of unhealthy behaviors that could impact their professional performance. Higher education institutions should establish health-promoting activities involving students, faculty, administrative staff, support personnel, and their families, without reducing their role to the provision of educational services^29^. The aforementioned aspects align with the Healthy Universities strategy^30^-^33^ in the Argentine Republic, which defines such institutions as “those that promote the holistic health of their community by acting on the physical and social environment, the educational process, and the broader community in which they are embedded.”

**Limitations**

As limitations, the present study was conducted at a single public institution of higher education. Additionally, the specific characteristics of the study population (such as employment status) may not be representative of students in other national or international institutions. Another limitation lies in the use of self-reported measures to assess students’ lifestyles, which may result in response bias, as participants might report behaviors they perceive as ideal rather than those that accurately represent their behaviors. The sampling method used (convenience sampling) is also a limitation of the study, as it may have led to a possible selection bias.

The study highlights the use of an instrument validated in Argentina and widely employed, as well as the representativeness of the data among students in the nursing program. This work provides an updated background for the knowledge of self-care dynamics of Bachelor of Nursing students and how social, demographic, and academic variables influence them. Future studies should explore the impact of lifestyle on quality of life and academic performance, expand the sample size, compare public and private institutions, and identify the limitations to self-care in this population.

## Conclusions

The LS of the nursing students was categorized as regular, characterized by unhealthy behaviors such as low responsibility in managing personal health, high levels of physical inactivity, poor dietary habits, and inadequate stress management. Overweight and obesity affected half of the participants, and a correlation was found between BMI and scores in the Nutrition dimension.

Higher mean scores (indicating a healthier lifestyle) were found in the Health Responsibility dimension among women, in the Physical Activity dimension among students without children, those exclusively dedicated to studying, and those enrolled in the third year of the study plan. Likewise, better stress management was observed among students without children and those not working, whereas poorer nutritional habits were reported among students enrolled in the first year of the nursing program.
